# Structure–Activity Relationships in Ni-Al Mixed Oxides: The Critical Role of a Precursor Anion in the Oxidative Dehydrogenation of Ethane

**DOI:** 10.3390/molecules30173465

**Published:** 2025-08-22

**Authors:** Qingzhu Meng, Dongxu Han, Dong Li, Yang Dong, Yanrong Wang, Lian Kong, Wanli Kang, Saule B. Aidarova, Zhen Zhao

**Affiliations:** 1 School of Materials Science and Green Technologies, Kazakh-British Technical University, Almaty 050000, Kazakhstan; 15940510950@163.com; 2 Institute of Catalysis for Energy and Environment, College of Chemistry and Chemical Engineering, Shenyang Normal University, Shenyang 110034, China; hdx17824030075@163.com (D.H.); lidongsynu@163.com (D.L.); yanrongwang2024@126.com (Y.W.); 3 State Key Laboratory of Heavy Oil Processing, China University of Petroleum, Beijing 102249, China; dongyangcup@163.com

**Keywords:** Ni-Al mixed oxide, oxidative hydrogenation of ethane, ball milling, nickel precursor, structure-performance relationship

## Abstract

The study employed a green, template-free ball milling method to construct a series of Ni-Al mixed oxide catalysts modulated by different nickel precursors (nitrate, acetate, carbonate, sulfate, and chlorate). Through multiscale characterization techniques (XRD, TEM, XPS, H_2_-TPR, etc.) and catalytic performance evaluations, we systematically elucidated the regulatory mechanism of precursor types on the structure-performance relationship. The NiAlO_x_-CO_3_^2−^ catalyst derived from nickel carbonate exhibited a unique structure, an optimal Ni/Al ratio, and well-tuned active oxygen species, thereby demonstrating exceptional catalytic performance in the oxidative dehydrogenation of ethane (ODHE) at 475 °C with 53.2% ethane conversion, 72.6% ethylene selectivity, and maintained stability over 40 h of continuous operation. Beyond developing high-performance ODHE catalysts, this work establishes a “precursor chemistry–material structure–catalytic performance” relationship model, offering new insights for the rational design of efficient catalysts for light alkane conversion.

## 1. Introduction

As the most essential feedstock in modern chemical industry [1,2,3], ethylene (C_2_H_4_) has seen global demand exceeding 200 million tons annually, with a steady growth rate of 3.1% [4]. Although steam cracking remains the dominant production method, its operation under harsh conditions (1000–1200 K) results in tremendous energy consumption and carbon emissions [5]. Shale gas and natural gas have provided abundant ethane resources (up to 20 vol%), making the development of novel ethane conversion technologies strategically important. The oxidative dehydrogenation of ethane (ODHE) has attracted considerable attention due to its exothermic nature and milder conditions (carbon suppression); however, it suffers from the critical challenge of C_2_H_4_ selectivity loss caused by over-oxidation (C_2_H_6_/C_2_H_4_ + O_2_ → CO_x_ + H_2_O) [6,7,8,9,10]. Therefore, the development of highly efficient catalysts is crucial for enhancing C_2_H_4_ selectivity.

Although the oxidative dehydrogenation of ethane (ODHE) has achieved remarkable academic progress due to its relatively mild operation conditions [2,11,12], it has not yet been implemented in industrial applications. Current research on ODHE catalysts primarily focuses on transition metal oxides, such as Mo/V-based oxides and Ni-containing systems [13,14,15,16]. Although catalysts such as MoVTeNbO_x_ demonstrate outstanding performance with C_2_H_4_ yields of 60–75% [17,18], their complex composition and stringent preparation requirements hinder practical applicability. In contrast, Ni-based catalysts have emerged as promising alternatives due to their moderate C-H bond activation capability and cost-effectiveness; however, they still face the intrinsic challenge of balancing catalytic activity and C_2_H_4_ selectivity [4,6,7,19]. Breakthrough studies reveal that constructing Ni-M solid solutions (M = Nb, Sn, Ta, and W, etc.) can effectively modulate the distribution of active oxygen species, as exemplified by Ni_85_Nb_15_O_x_ catalyst achieving 46% C_2_H_4_ yield [19,20,21,22], attributed to the reduced non-selective oxygen concentration (O^−^) and oxygen mobility through high-valent metal doping. Notably, beyond doping strategies, synthesis methods critically determine the catalytic performance of Ni-based catalysts. While conventional methods such as sol–gel, co-precipitation, and the dry mixing method have been extensively investigated with respect to synthetic parameters and Ni/metal ratio, the essential role of precursor chemistry has been received insufficient attention. Although nickel nitrate hexahydrate (Ni(NO_3_)_2_·6H_2_O) is widely adopted, a systematic investigation into the effects of precursors on ODHE performance is still lacking. Intriguingly, in other catalytic systems (e.g., NiFe oxides for the oxygen evolution reaction [23] and zeolite-supported nickel catalysts for carbon oxide methanation [24], precursor anions have been proven to significantly influence the resulting phase composition, metal dispersion, and oxidation states, etc. Therefore, simply tuning nickel salt precursors to optimize Ni-M mixed oxides for ODHE not only facilitates efficient materials discovery but also provides new insights into formation mechanisms of the active site.

This study presents an innovative green mechanochemical synthesis strategy for preparing a series of Ni-Al mixed oxides by systematically varying the anions of nickel precursor (NO_3_^−^, CO_3_^2−^, Cl^−^, SO_4_^2−^, and acac^−^). The catalytic performance was rigorously evaluated for the ODHE reaction using O_2_ as the oxidant. Notably, the catalyst derived from the nickel carbonate precursor exhibited significantly enhanced C_2_H_6_ conversions while maintaining comparable C_2_H_4_ selectivities. A comprehensive suite of characterization techniques (XRD, XPS, H_2_-TPR, etc.) was conducted to elucidate the structure-property relationship and reveal the origins of performance differences. The green synthesis method offers several advantages: (1) solvent-free operation at room temperature with 100% atom efficiency; (2) simple procedure with strong scalability potential; (3) in situ construction of active sites during precursor decomposition. Beyond the development of high-performance ODHE catalysts, this study establishes a fundamental “precursor chemistry-catalyst structure-catalytic performance” correlation, providing new insights for designing efficient alkane conversion catalysts.

## 2. Results and Discussion

**Figure 1:**
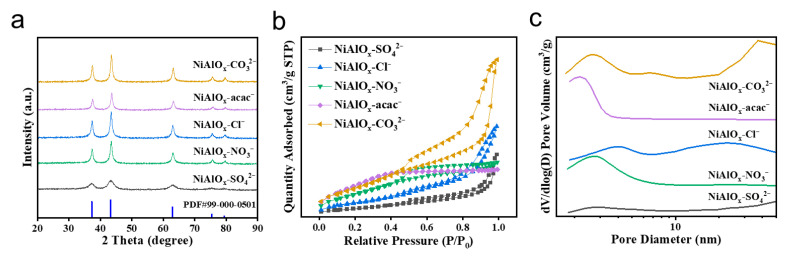
(**a**) XRD patterns, (**b**) N_2_ adsorption–desorption isotherms and (**c**) pore diameter curves of series NiAlO_x_-P catalysts.

The NiAlO_x_-P catalysts (P = NO_3_^−^, CO_3_^2−^, SO_4_^2−^, Cl^−^, and acac^−^) were successfully synthesized via a simple ball-milling method (details are provided in the Section 3). Multiscale characterizations systematically revealed the precursor-dependent structural evolution. The XRD patterns (Figure 1a) display characteristic peaks of cubic NiO (ICSD 9866) at 36.8°, 43.6° and 63.5°, corresponding to the (111), (200) and (220) crystallographic planes [9,25], respectively. Notably, all NiAlO_x_-P samples show consistent peak shifts toward higher diffraction angles (Δ2*θ* ≈ 0.2°) compared with pure NiO, which indicates successful incorporation of Al^3+^ ions into the NiO lattice (ionic radii: Al^3+^ = 0.53 Å vs. Ni^2+^ = 0.69 Å). However, the absence of detectable Al_2_O_3_ or NiAl_2_O_4_ phases suggests that Al species exist either as a Ni-Al solid solution or as highly dispersed phases in the NiO lattice [26]. Furthermore, the crystallinity of the materials is strongly dependent on the type of precursor used, with the NiCO_3_-derived sample exhibiting the sharpest (200) peak, whereas the NiSO_4_-derived sample shows the broadest diffraction peak.

N_2_ physisorption analyses (Figure 1b,c) reveal a precursor-dependent evolution of the pore structure. The NiAlO_x_-CO_3_^2−^, NiAlO_x_-Cl^−^, and NiAlO_x_-SO_4_^2−^ samples exhibit IV isotherms with H3 hysteresis loops, whereas the NiAlO_x_-NO_3_^−^ and NiAlO_x_-acac^−^ samples show IV isotherms with H4 hysteresis loops [4,6,7,27], suggesting the formation of two types of mesopores in these materials. The NiAlO_x_-CO_3_^2−^ sample possesses a higher surface area (212 m^2^/g) and pore volume (0.46 cm^3^/g), which can be attributed to in situ pore generation by CO_2_ release during precursor decomposition. The pore size distribution (Figure 1c) confirms the presence of typical mesopores (2–6 nm) across all samples, which are beneficial for efficient mass transport.

**Figure 2:**
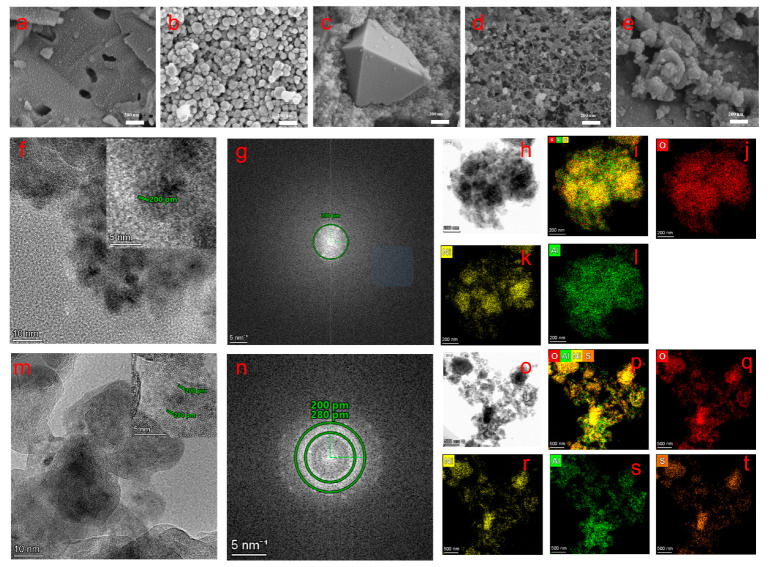
SEM images of (**a**) NiAlO_x_-acac^−^, (**b**) NiAlO_x_-NO_3_^−^, (**c**) NiAlO_x_-Cl^−^, (**d**) NiAlO_x_-CO_3_^2−^, and (**e**) NiAlO_x_-SO_4_^2−^. TEM images of (**f**) NiAlO_x_-CO_3_^2−^ and (**m**) NiAlO_x_-SO_4_^2−^. Forward Fourier Transform (FFT) images of (**g**) NiAlO_x_-CO_3_^2−^ and (**n**) NiAlO_x_-SO_4_^2−^. Typical TEM light-field images and elemental mapping images of (**h**–**l**) NiAlO_x_-CO_3_^2−^ and (**o**–**t**) NiAlO_x_-SO_4_^2−^. Red: O; yellow: Ni; green: Al; orange: S.

SEM analyses (Figure 2a–e) reveal the significant influence of Ni precursors on the morphology of NiAlO_x_-P materials. While all samples exhibit nanoparticle-based structures of varying sizes, the type of precursor anions distinctly directs the formation of specific morphologies. For instance, NiAlO_x_-NO_3_^−^ displays polydisperse nanoparticles ranging from 50 to100 nm in size, whereas NiAlO_x_-Cl^−^ forms well-defined octahedral structures with an edge length of approximately 800 nm, possibly attributed to Cl^−^-mediated facet-specific growth. Notably, NiAlO_x_-CO_3_^2−^ exhibits unique interconnected hierarchical pores, which may enhance its catalytic performance through a three-dimensional interconnected pore network [28].

TEM and STEM-EDS analyses (Figure 2f–t) revealed microstructural differences among the series of NiAlO_x_-P samples. NiAlO_x_-CO_3_^2−^ predominantly consists of a pure NiO phase with a lattice spacing of 0.20 nm (Figure 2g and inset in Figure 2f), along with homogeneous distribution of Ni, Al, and O elements (Figure 2h–l). In contrast, the TEM images in Figure 2m,n show that the NiAlO_x_-SO_4_^2−^ sample exhibits a biphasic structure composed of NiO and residual NiSO_4_ (lattice spacing of 0.28 nm). Elemental mapping confirms the incomplete decomposition of the NiSO_4_ precursor, resulting in the presence of S element (Figure 2o–t). Such structural divergence originates from their different thermal decomposition behaviors—nickel carbonate fully decomposes during the calcination process, whereas nickel sulfate remains partially undecomposed due to its higher thermal stability.

**Figure 3:**
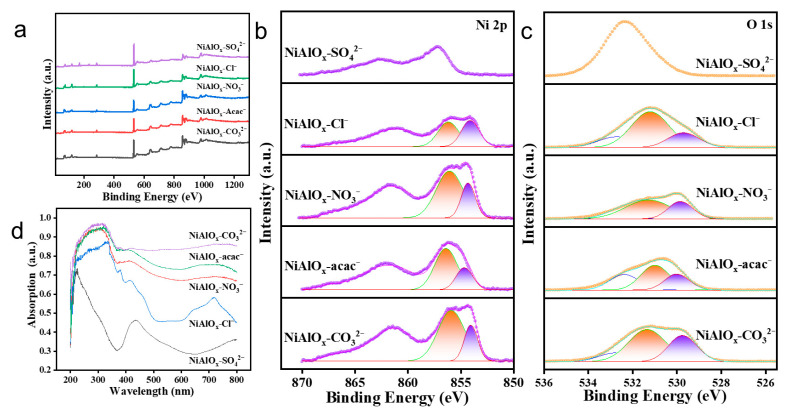
(**a**) XPS survey spectra, (**b**) Ni 2p XPS spectra, and (**c**) O 1s XPS spectra and (**d**) UV-Vis DRS spectra of the NiAlO_x_-P catalysts.

XPS survey spectra (Figure 3a) confirm that all NiAlO_x_-P catalysts except for NiAlO_x_-SO_4_^2−^ consist of Ni, Al, and O elements. Deconvolution of Ni 2p spectra (Figure 3b) revealed: (i) a main peak at 854.3 eV assigned to stoichiometric Ni^2+^ in NiO lattice, and (ii) a satellite peak (Sat I) at 855.9 eV corresponding to non-stoichiometric species (Ni^3+^, Ni^2+^-OH and cation vacancies). The peak area ratio of the Sat I/main peak serves as an indicator of catalyst non-stoichiometry, with a higher ratio indicating a greater extent of non-stoichiometry. Among the NiAlO_x_-P catalysts prepared from different Ni sources, NiAlO_x_-CO_3_^2−^ displays the highest value (ratio = 2.84). Notably, the characteristic peak at 857 eV in NiAlO_x_-SO_4_^2−^ provides evidence for the presence of a residual NiSO_4_ phase, likely resulting from incomplete decomposition of Ni precursor. O 1s spectral deconvolution (Figure 3c) shows three peaks for all NiAlO_x_-P catalysts except NiAlO_x_-SO_4_^2−^: 529.7 eV (lattice oxygen, O_L_), 531.4 eV (defective oxygen, O_V_), and 533.7 eV (surface-adsorbed Ni-OH). By comparison, it can be observed that the binding energy values of catalysts prepared from different nickel sources exhibit slight shifts, suggesting that nickel precursor engineering can tailor surface oxygen distribution. Generally, the abundance of O_L_ governs C_2_H_4_ selectivity, whereas O_V_ derived from Ni^3+^-O interactions and surface O^−^ species acts as the key active oxygen species for ethane activation and predominantly influences C_2_H_6_ conversion. The O_V_/O_L_ ratio analysis confirms that NiAlO_x_-CO_3_^2−^ has the highest ratio (0.62), indicating a maximized O_V_ content and suggesting the potential for higher ethane conversion. In contrast, the O_V_/O_L_ ratio of NiAlO_x_-Cl^−^ is the lowest (0.34). However, its abundant O_L_ species may result in a relatively lower ethane conversion rate while promoting higher ethylene selectivity.

UV-Vis diffuse reflectance spectroscopy (UV-Vis DRS, Figure 3d) was employed to analyze the electronic structures of NiAlO_x_-P catalysts obtained from various nickel precursors. The characteristic absorption peaks observed in all NiAlO_x_-P catalysts appear within the range of 300–400 nm, which correspond to the *d-d* electronic transitions of octahedrally coordinated Ni^2+^ species in the NiO lattice [29,30]. Significant variations in absorption intensity are observed among the samples. NiAlO_x_-CO_3_^2−^ exhibits the strongest absorption intensity, indicating its superior capability for electron transition, potentially leading to enhanced ethane conversion. In contrast, NiAlO_x_-SO_4_^2−^ shows remarkably lower absorption intensity, likely due to residual undecomposed NiSO_4_ resulting from its relatively high thermal stability. This residue sulfate may hinder electron transfer and consequently reduce the catalytic activity of the catalyst.

**Figure 4:**
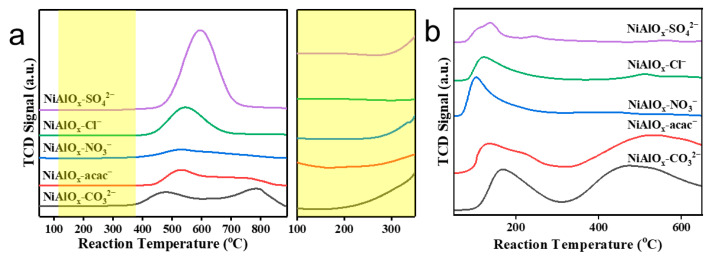
(**a**) H_2_-TPR and (**b**) NH_3_-TPD spectra of the NiAlO_x_-P catalysts.

The ODHE reaction over Ni-based catalysts proceeds via a redox mechanism, in which the heterolytic cleavage of C-H bonds on the NiO surface represents the rate-determining step. Consequently, the reduction behavior of NiO significantly governs its ODHE performance. H_2_-TPR analysis was conducted to investigate the redox properties of NiAlO_x_-P catalysts synthesized using different nickel precursors. As depicted in Figure 4a, NiAlO_x_-CO_3_^2−^ shows an initial reduction at ~250 °C, indicating the presence of surface non-stoichiometric Ni species. Its main reduction peaks appear at 469 °C and 786 °C, corresponding to the reduction of bulk Ni-O Ni(II) and Ni-O-Al Ni(II) species, respectively [31]. NiAlO_x_-NO_3_^−^ and NiAlO_x_-acac^−^ exhibit analogous reduction profiles but show markedly reduced intensity in the high-temperature peak. In contrast, NiAlO_x_-Cl^−^ and NiAlO_x_-SO_4_^2−^ display only a single intense reduction peak at 544 °C and 593 °C, respectively, which can be assigned to the reduction of bulk Ni-O species. These findings demonstrate that Ni precursor selection can precisely tune both the active oxygen species and the Ni-Al interactions in NiAlO_x_-P catalysts.

The acid–base properties of catalysts critically influence reactant adsorption and product desorption. Appropriate acidic sites can effectively facilitate C-H bond activation in ethane. NH_3_-TPD analysis (Figure 4b) further correlated acidic properties with C-H activation capability. All catalysts show weak acid sites in the temperature range of 50–300 °C, with NiAlO_x_-CO_3_^2−^ exhibiting the strongest acidity, thereby promoting initial ethane adsorption. In the high-temperature region (>300 °C), distinct desorption peaks associated with medium-strength acid sites are observed exclusively for NiAlO_x_-CO_3_^2−^ and NiAlO_x_-acac^−^, which can effectively polarize C-H bonds [32]. Conversely, other samples lack medium-strength acid sites, resulting in limited C-H bond activation ability. The combined analysis of H_2_-TPR and NH_3_-TPD results indicates that NiAlO_x_-CO_3_^2−^ possesses superior potential for ethane C-H bond activation.

**Figure 5:**
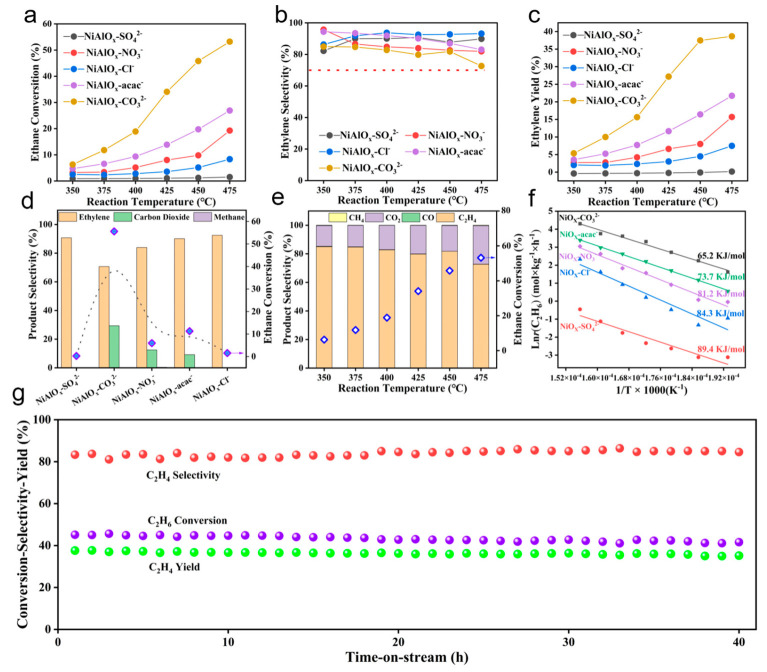
(**a**) Ethane conversion, (**b**) ethylene selectivity, and (**c**) ethylene yield as a function of reaction temperature (reaction conditions: *F* = 30 mL/min, C_2_H_6_/O_2_ = 1/1). (**d**) Corresponding product selectivity and ethane conversion over series NiAlO_x_-P catalysts at 475 °C. (**e**) Product distribution over the NiAlO_x_-CO_3_^2−^ catalyst. (**f**) Ln*r*(C_2_H_6_) versus 1/T over series NiAlO_x_-P catalysts. (**g**) Ethane conversion, selectivity and yield of ethylene over the NiAlO_x_-CO_3_^2−^ catalyst as a function of the time-on-stream.

The influence of nickel precursors on the ODHE performance was systematically evaluated over NiAlO_x_-P catalysts. As depicted in Figure 5a–d, ethane conversion increases markedly with reaction temperature for all catalysts (except NiAlO_x_-SO_4_^2−^), indicating enhanced C-H bond activation at elevated temperatures. It is noteworthy that NiAlO_x_-CO_3_^2−^ exhibits higher ethane conversions (53.2% at 475 °C) compared with other catalysts. Comparative analysis reveals that NiAlO_x_-CO_3_^2−^ achieves a 77-fold higher ethane conversion than NiAlO_x_-SO_4_^2−^ at 475 °C, underscoring the critical role of nickel precursors. Although the ethylene selectivities over NiAlO_x_-CO_3_^2−^ catalyst are slightly lower than those of other NiAlO_x_-P catalysts, it still remains above 70% at 475 °C (Figure 5b). The ethylene yield reaches 38.7%, surpassing most reported Ni-Al-O catalysts (Figure 5c) [4,6,7]. Moreover, it is evident that the notable increase in ethane conversion over the NiAlO_x_-CO_3_^2−^ catalyst does not result in a significant reduction in ethylene selectivity (Figure 5d). The enhancement in catalytic activity may originate from the CO_3_^2−^-induced optimization of active oxygen distribution, as well as the modulation of redox properties and acid-base characteristics, all of which collectively facilitated the selective cleavage of C-H bonds. Product distribution (Figure 5e) further demonstrates that ethylene and CO_2_ dominate the products over NiAlO_x_-CO_3_^2−^, with trace CH_4_ only at high temperatures, verifying the preferential oxidative dehydrogenation pathway over cracking. The kinetic studies (Figure 5f) reveal substantially lower apparent activation energy for NiAlO_x_-CO_3_^2−^ (65.2 kJ/mol) versus NiAlO_x_-SO_4_^2−^ (89.4 kJ/mol), directly correlating with its enriched active oxygen species (verified by XPS and H_2_-TPR) and optimized acid sites (confirmed by NH_3_-TPD). The 40 h continuous reaction experiment demonstrates that the NiAlO_x_-CO_3_^2−^ catalyst has excellent stability under reaction conditions (Figure 5g). Collectively, nickel precursor engineering allows for the modulation of physicochemical properties and the controlled distribution of reactive oxygen species, thereby enabling targeted optimization of ODHE performance over Ni-Al mixed oxides.

**Figure 6:**
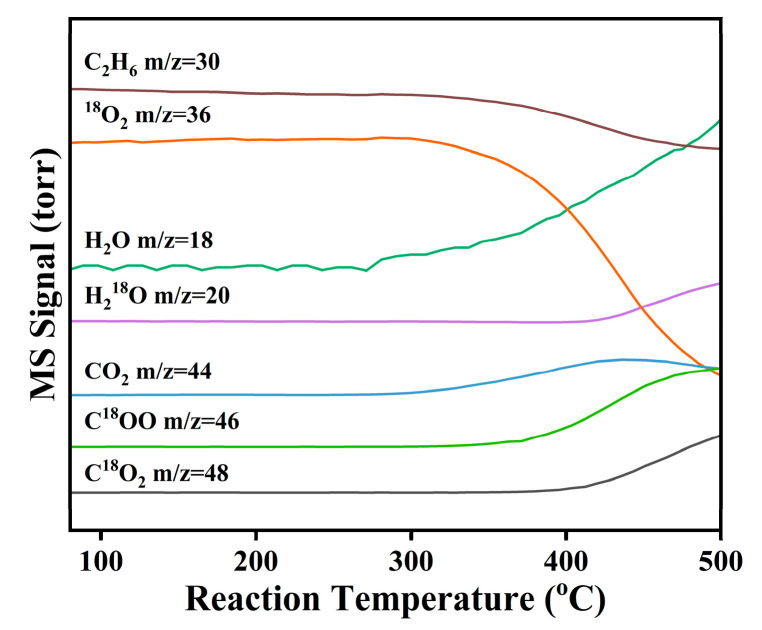
^18^O_2_-TPSR results over NiAlO_x_-CO_3_^2−^ catalyst. Reaction conditions: 1 vol% C_2_H_6_/1 vol% ^18^O_2_/N_2_ and *F* = 30 mL/min.

Mechanistic insights were gained through a ^18^O_2_ isotope-labeled temperature-programmed surface reaction (^18^O_2_-TPSR) experiment on the NiAlO_x_-CO_3_^2−^ catalyst, with results shown in Figure 6. Under co-feeding of ^18^O_2_ and C_2_H_6_, ethane conversion begins at approximately 250 °C. In the initial reaction stage after introducing of ^18^O_2_, H_2_^16^O (*m*/*z* = 18) and C^16^O_2_ (*m*/*z* = 44) are predominantly generated, confirming the involvement of lattice oxygen (^16^O_L_) in the initial C-H activation. Subsequently, signals corresponding to H_2_^18^O (*m*/*z* = 20) and C^16^O^18^O (*m*/*z* = 46) emerge and gradually intensify, demonstrating gaseous ^18^O_2_ incorporation into the reaction pathway. Crucially, the production of C^18^O_2_ is distinctly delayed. These observations clearly indicate that ^16^O_L_ is the primary active oxygen species participating in the ODHE reaction, whereas gaseous ^18^O_2_ functions to replenish the oxygen vacancies (O_V_) to complete the overall redox cycle (C_2_H_6_ + ^16^O_L_ → C_2_H_4_ + H_2_^16^O + O_V_, O_V_ + 1/2 ^18^O_2_ → ^18^O_L_). Taken together, these findings confirm that ethane ODH over the NiAlO_x_-CO_3_^2−^ catalyst proceeds via a classical Mars–van Krevelen (MvK) redox mechanism [33,34].

**Figure 7:**
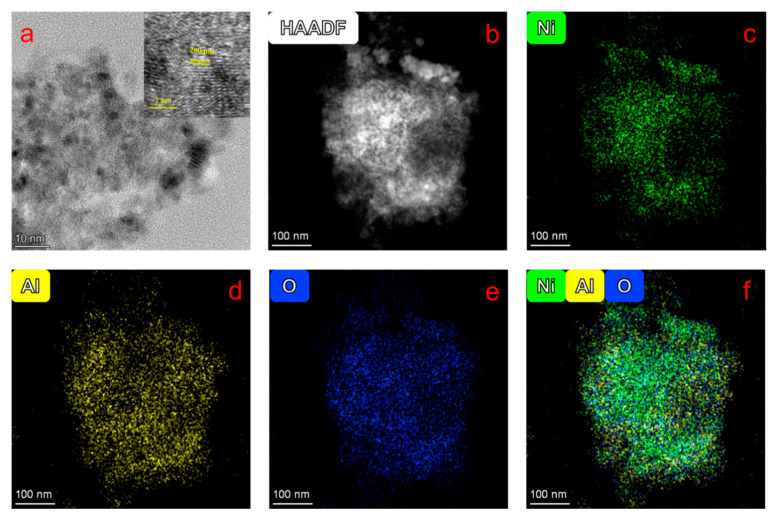
(**a**) TEM (**b**) HAADF and (**c**–**f**) elemental mapping images of the NiAlO_x_-CO_3_^2−^ catalyst after the ODHE reaction.

Atomic-scale characterization of the spent NiAlO_x_-CO_3_^2−^ catalyst is presented in Figure 7. TEM (Figure 7a) and HRTEM (inset of Figure 7a) images reveal a well-preserved lattice structure with an interplanar spacing of 0.20 nm corresponding to the (200) plane. HAADF and elemental mapping images (Figure 7b–f) demonstrate a homogeneous distribution of Ni (green), Al (yellow), and O (blue) at nanoscale, without evidence of phase segregation or metal aggregation. These results, combined with 40 h stability test, validate outstanding thermal and structural stability of the catalyst.

**Figure 8:**
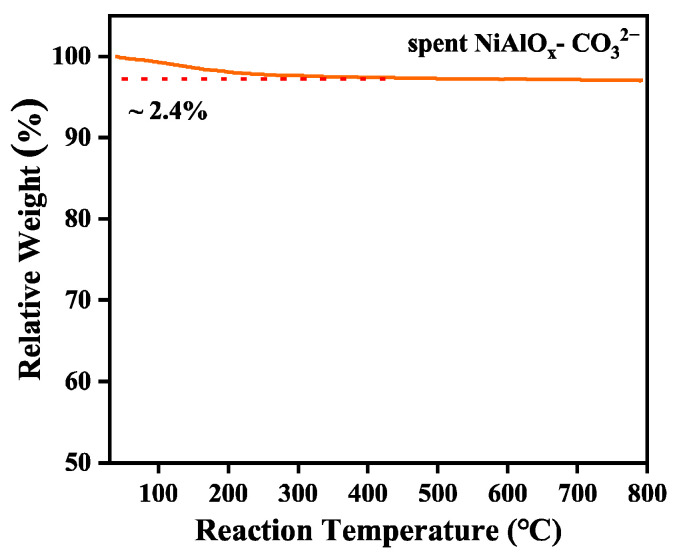
TGA curve of after the NiAlO_x_-CO_3_^2−^ catalyst after the ODHE reaction.

Carbon deposition on the spent NiAlO_x_-CO_3_^2−^ catalyst was quantitatively analyzed by thermogravimetric analysis (TGA) under an air atmosphere. As shown in the TAG curve (Figure 8), only a 2.4% mass loss is observed below 250 °C, which is attributed to the desorption of physically adsorbed water. No significant weight-loss steps associated with carbon oxidation are detected during further heating up to 800 °C. Combined with the post-reaction TEM images (Figure 7), the result confirms the material’s exceptional resistance to coking.

## 3. Materials and Methods

### 3.1. Catalyst Preparation

Nickel precursors, including nickel carbonate (NiCO_3_), nickel acetylacetonate (Ni(acac)_2_), nickel sulfate (NiSO_4_), nickel nitrate hexahydrate (Ni(NO_3_)_2_·6H_2_O), and nickel chloride hexahydrate (NiCl_2_·6H_2_O), were obtained from Sinopharm Chemical Reagent Co., Ltd. (Shanghai, China). Aluminum isopropoxide (Al(O-*i*-Pr)_3_, Sinopharm Chemical Reagent Co., Ltd.) was used as the aluminum source. All chemicals were of analytical grade and were used without further purification.

Ni-Al mixed oxides were synthesized via a mechanochemical route. Al(O-*i*-Pr)_3_ and different nickel precursors were precisely weighed based on a fixed Al/Ni molar ratio of 0.8:1. The mixtures were subjected to ball milling at 350 rpm for 120 min under ambient temperature, aiming to achieve molecular-level homogenization. Subsequently, the resulting mixtures were dried at 80 °C for 12 h, followed by calcination in static air with a heating rate of 1 °C/min up to 550 °C for holding 6 h. The obtained catalysts were denoted as NiAlO_x_-P (P = CO_3_^2−^, NO_3_^−^, SO_4_^2−^, Cl^−^, and acac^−^).

### 3.2. Characterization

X-ray diffraction (XRD) patterns were analyzed on a Rigaku Ultima IV diffractometer (Rigaku, Tokyo, Japan) with Cu K*α* radiation (40 kV, 40 mA) over a 2*θ* range of 10–90° at a scanning rate of 5 ^o^/min. N_2_ physisorption at −196 °C was performed on a Micromeritics TriStar II 3020 analyzer (Micromeritics, Norcross, GA, USA). Prior to measurement, the samples were degassed under vacuum at 300 °C for 3 h. The pore size distribution was analyzed using the BJH model, while the BET equation was employed to calculate the specific surface area. UV-Vis diffuse reflectance (UV-Vis DRS) spectra were recorded on a Hitachi (Tokyo, Japan) UH4150 spectrometer in the wavelength range of 200–800 nm with a scan rate of 120 nm/min. BaSO_4_ was employed as the reference material. Morphology was characterized using field-emission scanning electron microscopy (SEM, ZEISS Gemini 300, Oberkochen, Germany) at an accelerating voltage of 3.0 kV. Transmission electron microscopy (TEM) images and energy dispersive X-ray (EDX) mapping were acquired on a Thermo Fisher Scientific (Waltham, MA, USA) FEI Talos F200X microscope operated at 200 kV. X-ray photoelectron spectroscopy (XPS) measurements were carried out using a Thermo Fisher Scientific spectrometer equipped with an Al K*α* excitation source (*hv* = 1486.6 eV). The binding energies were calibrated to the C 1s peak of contamination carbon at 284.8 eV. Shirley background subtraction was applied, and peak deconvolution was performed using the mixed Gaussian-Lorentzian (GL) functions with a 30% Lorentzian component. The fitting constraints included a full width at half maximum (FWHW ≤ 1.5 eV) for all peaks and a fixed spin–orbit splitting of 17.8 eV for the Ni 2p_3/2_-2p_1/2_ doublet. Spectral fitting was optimized using Origin 2017 software, and the peak assignments were validated against reference spectra from NiO (ICSD 9866) and NiAl_2_O_4_ (ICSD 00-010-0339). H_2_ temperature-programmed reduction (H_2_-TPR) analysis was conducted on a Xianquan (Changzhou, China) TP-5080-D analyzer. 100 mg sample was first pretreated in Ar at 300 °C for 30 min, followed by heating to 900 °C (10 °C/min) under a 10% H_2_/Ar flow (30 mL/min). Ammonia temperature-programmed desorption (NH_3_-TPD) was conducted using the same equipment as H_2_-TPR. 100 mg sample was degassed under He at 300 °C for 30 min, then heated to 900 °C (10 °C/min) with desorbed species monitored by TCD. Carbon deposition was quantified using a thermogravimetric system (TG) (NETZSCH STA449 F5, Selb, Germany). The analysis was conducted under an air atmosphere at 10 °C/min to 900 °C, with CO_2_ (*m*/*z* = 44) and H_2_O (*m*/*z* = 18) monitored via mass spectrometry (MS). ^18^O_2_ isotopic temperature-programmed surface reaction (^18^O_2_-TPSR) experiment was performed in a fixed-bed reactor (HP WF51) coupled with a Hiden MS. Prior to the reaction, 100 mg catalyst was activated under an Ar atmosphere at 300 °C for 1 h. Subsequently, the catalyst was exposed to C_2_H_6_/^18^O_2_/Ar mixture (3:3:24 *v*/*v*, 30 mL/min), while the temperature was gradually increased from 50 °C to 650 °C (10 °C/min). The following *m*/*z* values were monitored as key species: 30 (C_2_H_6_), 28 (C_2_H_4_), 2 (H_2_), 32 (^16^O_2_), 12 (CO), 18 (H_2_^18^O), 20 (H_2_^18^O), 44 (C^16^O_2_), 46 (C^16^O^18^O), 48 (C^18^O_2_), 36 (^18^O_2_).

### 3.3. Catalyst Evaluation

The ODHE performance was evaluated in a fixed-bed reactor (HPWF-51, Nanjing Hope analysis equipment Co., Ltd., Nanjing, China) at atmospheric pressure. A 100 mg catalyst (60–80 mesh) was loaded into a quartz tube reactor and secured with quartz wool. The reaction gas mixture (C_2_H_6_:O_2_:N_2_ = 3:3:24 *v*/*v*) was introduced at a gas hourly space velocity (GHSV) of 18,000 mL/(g·h), with individual flow rates controlled by mass flow controllers (Beijng Sevenstar Flow Co., Ltd., Beijing, China). Reaction temperature was programmed from 350 °C to 475 °C, with a temperature control accuracy of ±1 °C. Reactants and products were analyzed using an online gas chromatograph (Agilent 7890B, Santa Clara, CA, USA). Hydrocarbons (C_2_H_6_, C_2_H_4_) were separated by an HP-FFAP capillary column and quantified with a flame ionization detector (FID), while permanent gases (O_2_, CO, and CO_2_) were separated using MolSieve 5A, HP Plot Q, and Porapark Q columns and detected by a thermal conductivity detector (TCD).

C_2_H_6_ conversion, C_2_H_4_ selectivity and yield were calculated according to Equations (1)–(3):

(1) $$C_{2}H_{6}\;\mathrm{conversion}\;(\%)\;=\;\frac{C_{2}H_{6\;}\mathrm{moles}\;\mathrm{of}\;(C_{2}H_{6,\;\mathrm{in}}-C_{2}H_{6,\;\mathrm{out}})}{C_{2}H_{6\;}\mathrm{moles}\;\mathrm{of}\;C_{2}H_{6,\;\mathrm{in}}}\;\times \;100\%$$

(2) $$C_{2}H_{4}\;\mathrm{selectivity}\;(\%)=\frac{\mathrm{moles}\;\mathrm{of}\;C_{2}H_{4}\;}{C_{2}H_{6\;}\mathrm{moles}\;\mathrm{of}\;(C_{2}H_{6,\;\mathrm{in}}-C_{2}H_{6,\;\mathrm{out}})}\;\times \;100\%$$

C_2_H_4_ yield (%) = (C_2_H_6_ conversion × C_2_H_4_ selectivity) × 100% (3)

## 4. Conclusions

This study presented a solvent-free mechanochemical strategy for fabricating high-performance NiAlO_x_-P catalysts modulated by nickel precursors (CO_3_^2−^, SO_4_^2−^, Cl^−^, NO_3_^−^, and acac^−^). This approach integrated green synthesis with precise structural control, thereby significantly improving the ODHE performance of Ni-Al mixed oxides. Characterization results revealed that precursor anions precisely regulate the microstructure, distribution of active oxygen species, and acid-base properties of the catalysts. Notably, the carbonate-derived NiAlO_x_-CO_3_^2−^ catalyst exhibited an exceptional performance, achieving 53.2% ethane conversion with 72.6% ethylene selectivity at 475 °C, while maintaining stable performance for 40 h. The enhanced performance can be attributed to (1) the optimization of active oxygen species distribution and adjustment of acid site density by CO_3_^2−^ ions; and (2) the strengthened Ni-Al interactions facilitating lattice oxygen mobility. Beyond providing a green and scalable synthesis route, this work establishes a “precursor chemistry–catalytic performance” relationship, offering fundamental insights into the rational design of efficient alkane dehydrogenation catalysts.

## Data Availability

The original contributions presented in this study are included in the article, Further inquiries can be directed to the Corresponding authors.
